# Sofosbuvir–velpatasvir single-tablet regimen administered for 12 weeks in a phase 3 study with minimal monitoring in India

**DOI:** 10.1007/s12072-019-09927-6

**Published:** 2019-02-21

**Authors:** Ajit Sood, Ajay Duseja, Mayank Kabrawala, Pradeep Amrose, Bhadadev Goswami, Abhijit Chowdhury, Shiv Kumar Sarin, Abraham Koshy, Robert H. Hyland, Sophia Lu, Gregory Camus, Luisa M. Stamm, Diana M. Brainard, G. Mani Subramanian, Madhura Prasad, Shobna Bhatia, Samir R. Shah, Dharmesh Kapoor, Shalimar, Vivek Saraswat

**Affiliations:** 1 Head of Department of Gastroenterology, Dayanand Medical College and Hospital, 6-E, Tagore Nagar, Ludhiana, 141001 India; 2 Post-graduate Institute of Medical Education and Research, Chandigarh, India; 3 Surat Institute of Digestive Sciences, Surat, India; 4 YRG CARE, Taramani, Chennai, India; 5 Institute of Digestive and Liver Disease, Ganeshguri, India; 6 Institute of Post Graduate Medical Education and Research, Kolkata, India; 7 Institute of Liver and Biliary Sciences, New Delhi, India; 8 Lakeshore Hospital, Kochi, India; 9 Gilead Sciences, Inc, Foster City, CA USA; 10 VGM Hospital, Coimbatore, India; 11 King Edward Memorial Hospital, Mumbai, India; 12 Global Hospital, Mumbai, India; 13 Global Hospital, Hyderabad, India; 14 All India Institute of Medical Science, Delhi, India; 15 Sanjay Gandhi Postgraduate Institute of Medical Sciences, Lucknow, India

**Keywords:** NS5B polymerase inhibitor, NS5A inhibitor, Public health, Pangenotypic

## Abstract

**Background and aims:**

In clinical studies, sofosbuvir–velpatasvir has demonstrated high cure rates and favorable tolerability in patients chronically infected with chronic hepatitis C virus (HCV) of any genotype. We evaluated the effectiveness and safety of sofosbuvir–velpatasvir administered with minimal medical monitoring to patients in India.

**Methods:**

At 16 sites in India, 129 adult patients with chronic HCV infection of any genotype initiated 12 weeks of once-daily sofosbuvir–velpatasvir (400–100 mg). Patients with compensated cirrhosis or prior treatment experience could be included in the study. Study drug was dispensed monthly, but there were no on-treatment study assessments. The primary efficacy endpoint was rate of sustained virologic response (HCV RNA < 15 IU/mL) 12 weeks after treatment (SVR12), which was compared to a pre-specified performance goal of 85%.

**Results:**

The majority of patients had HCV genotype 3 infection (70%), followed by HCV genotype 1 (22%). The SVR12 rate was 93% (120/129; 95% CI, 87% to 97%) (*p* = 0.009 compared with the 85% performance goal). Of the nine patients who did not achieve SVR12, 1 experienced virologic failure, 2 relapsed after treatment, 1 withdrew consent after treatment, and 5 were lost to follow-up (1 during and 4 after treatment). Sofosbuvir–velpatasvir was well-tolerated, and no patients discontinued treatment because of an adverse event. The most frequently reported adverse events were headache (3% of patients), upper abdominal pain (2%), and pyrexia (2%).

**Conclusions:**

In this study conducted at multiple sites in India, sofosbuvir–velpatasvir administered without genotype restriction or on-treatment safety assessments was well-tolerated and highly effective.

## Introduction

In India, the burden of chronic hepatitis C virus (HCV) infection is large [1]. An estimated 0.8% [2] of 1.3 billion persons are anti-HCV positive. Studies of subpopulations in India suggest prevalence rates vary widely (0.09–5.2%) [1, 3–9], although some of this variation can be attributed to differences in sampling technique. The majority of HCV-infected persons in India have genotype 3 virus [1, 2], which has been associated with a relatively aggressive course of liver disease, with increased risk of severe steatosis, fibrosis progression, or hepatocellular carcinoma [10, 11]. Relative to other genotypes, genotype 3 HCV has been less responsive to treatment with earlier-generation direct-acting antivirals [12] and requires longer durations of treatment or the addition of ribavirin, which is associated with anemia and therefore necessitates careful monitoring of patients during treatment.

The combination of sofosbuvir, NS5B polymerase inhibitor, with velpatasvir, NS5A inhibitor, is a once-daily, oral, pangenotypic, single-tablet regimen that is well-tolerated and leads to high SVR rates (95–99%) in patients with or without compensated cirrhosis in clinical studies [13, 14]. A key advantage of sofosbuvir–velpatasvir relative to earlier regimens containing direct-acting antivirals is that it can be used without ribavirin in all HCV genotypes and patients with compensated cirrhosis. The safety and tolerability of sofosbuvir–velpatasvir suggest it could be administered with minimal safety monitoring, an important consideration for regions with limited medical resources. We evaluated the safety and effectiveness of sofosbuvir–velpatasvir administered with no genotypic restrictions or on-treatment study assessments at multiple medical centers in India.

## Methods

### Patients

Eligible patients were adults aged 18 years or older with chronic HCV infection of any genotype. Patients could be treatment-naive or -experienced, and those without cirrhosis or with compensated cirrhosis were eligible for participation. The presence of cirrhosis was determined by either (1) liver biopsy with Metavir 4 or Ishak ≥ 5 scoring; (2) Fibroscan > 12.5 kPa; (3) abdominal ultrasound or radiographic imaging; or 4) laboratory tests such as FibroTest (> 0.75), AST:platelet ratio index (> 2), FIB-4 (> 5.88), or another test meeting accepted standards. Patients were excluded from participating in the study if they had any of the following conditions: platelets < 30,000/µL, hemoglobin < 8 g/dL, alanine aminotransferase or aspartate aminotransferase > 10 × ULN; creatinine clearance < 30 mL/min (Cockcroft–Gault equation); infection with hepatitis B or HIV; prior liver transplantation; hepatocellular carcinoma; or had previously been treated with a HCV non-structural (NS) protein 5A inhibitor. Patients provided written informed consent before undertaking any study-related procedures.

### Study design

This was an open-label, Phase 3 study. Up to approximately 20% of patients could have compensated cirrhosis, and up to approximately 20% could be treatment-experienced. Patients received sofosbuvir–velpatasvir (400–100 mg) combination tablet once-daily for 12 weeks. Study visits were conducted at screening, on day 1 of treatment, and at the end of treatment. In addition, drug-dispensing visits occurred at weeks 4 and 8. After completing 12 weeks of treatment, patients underwent follow-up visits at post-treatment weeks 4 and 12.

### Study oversight

The study protocol was approved by the review board or ethics committee of each institution prior to study initiation. The study was conducted in accordance with the International Conference on Harmonization Good Clinical Practice Guidelines and the Declaration of Helsinki.

### Assessments

Screening assessments included measurement of plasma HCV RNA level, HCV genotyping, and standard laboratory and clinical tests. HCV RNA levels were quantified by using the COBAS Ampliprep/COBAS TaqMan HCV Test, version 2.0 (Roche Molecular Systems, Inc., Branchburg, NJ), which has a lower limit of quantitation (LLOQ) of 15 IU/mL. HCV genotype and subtype were determined using the LiPA 2.0 genotyping assay or by Sanger sequencing if the LiPA assay did not provide a genotype.

Plasma HCV RNA levels were evaluated at screening, on day 1 of treatment, at the end of treatment, and at the week 12 follow-up visit. Plasma samples for viral sequencing were collected on day 1 of treatment, at the end of treatment, and at post-treatment week 12. Deep sequencing of the NS5A, and NS5B coding regions was performed on samples obtained from patients with virologic failure at baseline and at the first postbaseline time point during which a plasma sample with HCV RNA >1000 IU/mL was available. Sequences obtained from postbaseline and baseline samples were compared to detect treatment-emergent resistance-associated substitutions (RASs). Reported RASs were present in more than 15% of the sequence reads.

Physical examinations were conducted at screening, on treatment day 1, at the final treatment visit, and at the week 4 follow-up visit. Collection of vital signs, adverse events, concomitant medication intake, and clinical laboratory assessments followed the same schedule. Adverse events were coded using the Medical Dictionary for Regulatory Activities, Version 20.1. Study drug was dispensed monthly. Adherence study drug was measured by pill counts of bottles returned to the study site by patients. If a bottle was dispensed but not returned (missing), it was assumed that no study drug was taken from that bottle.

### Endpoints

The primary efficacy endpoint was achievement of SVR12, defined as having HCV RNA < LLOQ 12 weeks after discontinuing study drugs, among patients who took at least 1 dose of study drug.

### Statistical analyses

In the primary efficacy analysis, the SVR12 rate was compared to a pre-specified performance goal of 85% by using a 2-sided exact 1-sample binomial test at the 0.05 significance level. The basis for the 85% benchmark included the overall trend toward increasing SVR rates in recent years, the higher SVR rates observed with peg-interferon plus ribavirin in Asian patients [15], and the general appeal of using a fixed clinically relevant threshold as a measure of treatment benefit [16] of the pangenotypic regimen of sofosbuvir–velpatasvir.

A sample size of 125 patients was to provide 95% power to detect an improvement in SVR12 from 85% to 95% using a 2-sided exact 1-sample binomial test at a significance level of 0.05.

## Results

### Patient population

**Fig. 1:**
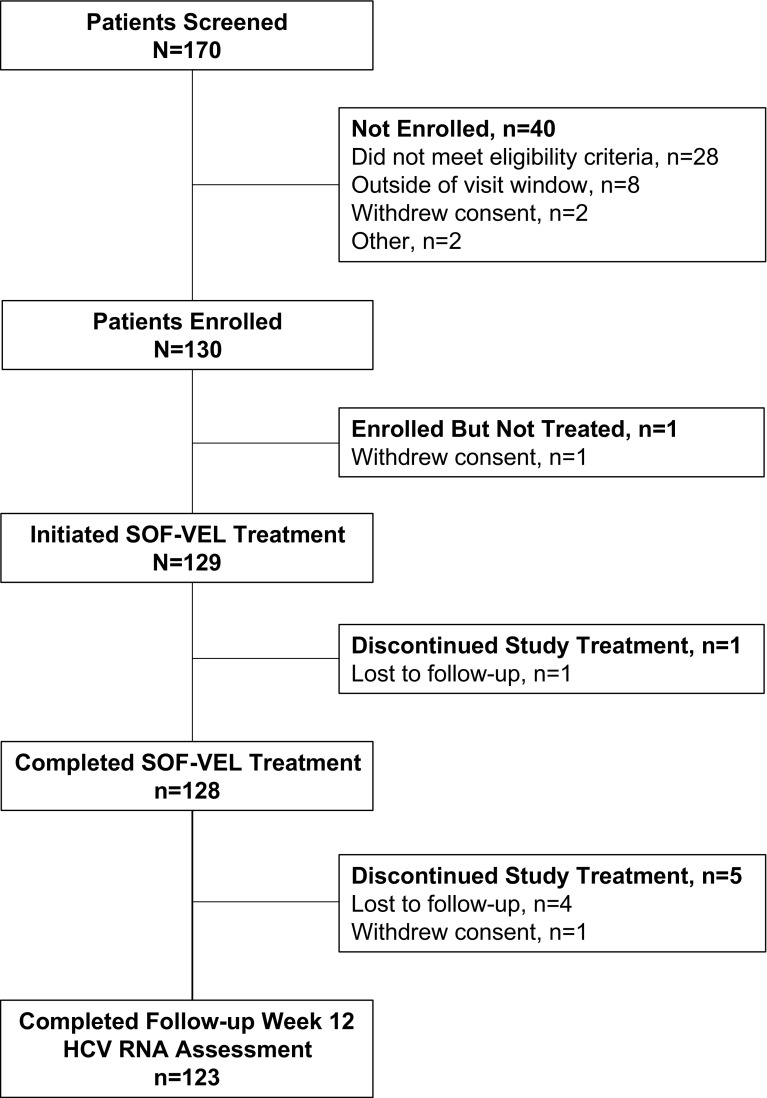
Patient disposition throughout treatment. *HCV* hepatitis C virus, *SOF* sofosbuvir, *VEL* velpatasvir

At 16 sites in 14 cities in India, 129 patients were enrolled and received at least 1 dose of sofosbuvir–velpatasvir. The majority of patients (59%) were male, and the median age was 42 years (range 19–75) (Table 1). Seventy-one percent of patients (90/129) were infected with HCV genotype 3. Forty-two patients (33%) had cirrhosis and 11 patients (9%) had prior treatment experience, 9 of whom (82%) had prior treatment with Peg-interferon and ribavirin. Of the 129 patients who initiated therapy, 128 (99%) completed it and 1 patient was lost to follow-up during treatment (Fig. 1).

**Table 1:** Patient demographics and baseline characteristics

	SOF-VEL 12 weeks (*N* = 129)
Median (range) age, year	42 (19–75)
Age < 65 years, *n* (%)	122 (95)
Male sex at birth, *n* (%)	76 (59)
Asian race, *n* (%)	129 (100)
Median (range) BMI, kg/m^2^	24 (15–40)
Genotype, *n* (%)	
1	28 (22)
1a	6 (5)
1b	22 (17)
3	90 (70)
3a	61 (47)
3b	11 (9)
3g	1 (1)
3i	6 (5)
3k	5 (4)
4	7 (5)
4a/4c/4d	1 (1)
4h	4 (3)
6c-1	1 (1)*
Indeterminate	3 (2)
Mean (SD) HCV RNA, log_10_ IU/mL	5.9 (0.96)
Cirrhosis, *n* (%)	42 (33)
Median (range) ALT, U/L	46 (11, 298)
Treatment experienced, *n* (%)	11 (9)
Peg-interferon + ribavirin	9 (7)

*ALT* alanine aminotransferase, *BMI* body mass index, *eGFR* estimated glomerular filtration rate, *HCV* hepatitis C virus, *SOF* sofosbuvir, *VEL* velpatasvir

*This patient was later determined to have HCV genotype 6n infection by BLAST analysis

### Efficacy

The SVR12 rate was 93% (120 of 129; 95% CI 87% to 97%) (Table 2), which was significantly higher than the prespecified performance goal of 85% (*p* = 0.009). Among patients with cirrhosis, 98% (41 of 42) achieved SVR12 (Table 3). Of the 11 patients who had previously received treatment for HCV, 10 (91%) achieved SVR12. At the end of treatment, 99% (112 of 113) of patients who underwent evaluation had undetectable HCV RNA. Of the nine patients who did not reach SVR12, one experienced on-treatment virologic failure, two relapsed after treatment, one withdrew consent after completing treatment, and five were lost to follow-up (one during treatment and four after treatment). Six additional patients did not achieve SVR12 (five patients were lost to follow-up and one patient withdrew consent). Of the six patients who were lost to follow-up and withdrew, 4 had genotype 3a, 1 had genotype 1a, and 1 had genotype 1b HCV infection. The overall relapse rate was 2% (2 of 112 patients).

**Table 2:** Treatment response to sofosbuvir–velpatasvir

	SOF-VEL 12 weeks (*N* = 129)
HCV RNA < LLOQ (15 IU/mL), *n*/*n* (%)	
On treatment	
Week 12	112/113 (99)
After treatment	
Week 12 (SVR12)	120/129 (93)
95% CI	87% to 97%
Virologic failure, *n* (%)	
On treatment	1
Relapse	2

*HCV* hepatitis C virus, *LLOQ* lower limit of quantification, *SOF* sofosbuvir, *SVR12* sustained virologic response 12 weeks after treatment, *VEL* velpatasvir

**Table 3:** SVR12 by subgroups

	SOF-VEL 12 weeks (*N* = 129)
Cirrhosis	
Yes	98% (41/42)
No	91% (79/87)
HCV genotype	
1 (all subtypes)	93% (26/28)
1a	83% (5/6)
1b	96% (21/22)
3	93% (84/90)
4	100% (7/7)
6	0% (0/1)
Indeterminate	100% (3/3)
Prior treatment	
Naive	93% (110/118)
Experienced	91% (10/11)
Adherence to SOF-VEL	
< 80%	88% (7/8)
≥ 80%	93% (113/121)

*HCV* hepatitis C virus, *SOF* sofosbuvir, *SVR12* sustained virologic response 12 weeks after treatment, *VEL* velpatasvir, *VOL* voxilaprevir

The patient who experienced on-treatment virologic failure was a 30-year-old male with HCV genotype 3a who did not have cirrhosis, had HCV RNA 3,900,000 IU/mL at baseline and 25 IU/mL at the end of treatment. This patient was reported as being adherent to treatment and did not have NS5A or NS5B RASs at baseline or postbaseline.

The two patients that relapsed had genotype 3b and genotype 6n HCV infection as determined by BLAST analysis. The patient with genotype 3b infection had cirrhosis and had been enrolled with an exclusionary prior treatment history having received sofosbuvir and daclatasvir for 12 weeks. This patient had NS5A RASs A30K and L31M at baseline and relapse. No NS5A RASs emerged at relapse (NS5B sequencing was not available due to assay failure). The other patient who relapsed with genotype 6n HCV infection was initially identified as having genotype 6c-1 by LiPA at screening. This patient was treatment naive and did not have cirrhosis. This patient had NS5A RASs F28V and T93S and the NS5B RAS M289L at baseline and relapse. No NS5A or NS5B RASs emerged at relapse.

### Safety

Fifteen percent (19 of 129) of patients experienced an adverse event (Table 4). The most commonly reported adverse events overall were headache (3%), upper abdominal pain (2%), and pyrexia (2%). One serious adverse event, rectal hemorrhage, was reported, and this event was not considered related to study treatment. The event occurred 20 days after the end of treatment and resolved 12 days after onset. No patient had adverse events leading to premature discontinuation of treatment or to interruption of sofosbuvir–velpatasvir dosing. The only Grade 3 or 4 laboratory abnormalities that occurred in more than 1 patient were decreased hemoglobin (five patients), lymphocytes (three patients), and platelets (two patients) and increased bilirubin (two patients); none of these labs was considered clinically significant. Of the five patients with Grade 3 or 4 decreased hemoglobin, four had Grade 2 decreased hemoglobin and one had normal hemoglobin levels at screening and/or day 1 of treatment and all five had maximal decreases to Grade 3. Overall, hemoglobin was stable during the study with the mean (median) change from baseline of 0.0 (0.1) g/dL at week 12 and 0.1 (0.2) g/dL at post-treatment week 4.

**Table 4:** Adverse events and laboratory abnormalities

	SOF-VEL 12 weeks (*N* = 129)
No. (%) of patients with any adverse event	19 (15)
No. (%) of patients with a Grade 3 or 4 adverse event	1 (1)
No. (%) of patients with a serious adverse event	1 (1)
Adverse events leading to discontinuation of study drug, *n* (%)	0
Deaths, *n*	0
Adverse events in > 1% of patients, *n* (%)	
Headache	4 (3)
Abdominal pain upper	3 (2)
Pyrexia	3 (2)
Fatigue	2 (2)
Hyperchlorhydria	2 (2)
Nausea	2 (2)
Upper respiratory tract infection	2 (2)
Serious adverse events, *n* (%)	
Rectal hemorrhage	1 (1)
Laboratory abnormalities (Grade 3 or above), *n* (%)	
Hemoglobin, 70 to < 90 g/L or ≥ 4.5 g/L decrease from baseline	5 (4)
Lymphocytes	
350 to < 500/mm^3^	1 (1)
<350/mm^3^	2 (2)
Bilirubin, > 2.5 to 5.0 × ULN	2 (2)
ALT, > 10.00 × ULN	1 (1)
AST, > 5.00 to 10.00 ULN	1 (1)
Glucose, > 250 to 500 mg/dL	1 (1)
Platelets	
25,000 to < 50,000/mm^3^	1 (1)
< 25,000 mm^3^	1 (1)
White blood cells, 1000 to < 1500/mm^3^	1 (1)

*ALT* alanine transaminase, *AST* aspartate transaminase, *SOF* sofosbuvir, *ULN* upper limit of normal, *VEL* velpatasvir

## Discussion

In this study conducted at multiple sites in India, sofosbuvir–velpatasvir administered without genotype restriction or on-treatment safety assessments was well-tolerated and highly effective with a 93% SVR12 rate. Overall, 9 (7%) of 129 patients did not achieve SVR12. Of these, 1 patient had on-treatment virologic failure, and two patients relapsed. One of the patients who had relapsed had been previously treated with NS5A inhibitor-containing regimen (SOF and DCV for 12 weeks). None of the three patients with virologic failure had treatment-emergent resistance. The SVR12 rates were > 90% for all subtype groups with greater than 1 patient, including patients with treatment experience, cirrhosis and rare subtypes of genotype 3 that are often underrepresented in studies taking place in Western countries.

These efficacy findings are consistent with those observed in the global sofosbuvir–velpatasvir development program. In the Phase 3 ASTRAL-1 (GS-US-342-1138), ASTRAL-2 (GS-US-342-1139), and ASTRAL-3 (GS-US-342-1140) studies, the SVR12 rate of sofosbuvir–velpatasvir for 12 weeks in patients with genotype 1–6 HCV infection with and without compensated cirrhosis was 98% (1015 of 1035 patients) and the relapse rate was 1% (13 of 1032 patients) [13, 14]. Compared with the global Phase 3 program, the overall SVR12 rate in this study was slightly lower due to the number of patients who did not achieve SVR12 due to non-virologic reasons; the relapse rate was similar (2%).Overall, treatment with sofosbuvir–velpatasvir for 12 weeks in patients with chronic HCV infection was generally safe and well tolerated. The overall rate of AEs was lower (15%) than had been previously observed in the ASTRAL studies (69%–88%) perhaps due to the design of the current study having fewer on-treatment assessments [13, 14].

Sofosbuvir–velpatasvir has multiple advantages relative to earlier direct-acting antiviral regimens with regard to diagnostic testing and monitoring. Because of its pangenotypic activity, HCV genotype assessment prior to sofosbuvir–velpatasvir treatment is not required. The treatment algorithm is further simplified in the single treatment duration for all degrees of liver fibrosis and the absence of ribavirin greatly reduces the need for safety monitoring on treatment. During the conduct of this study, sofosbuvir–velpatasvir was approved in India and sofosbuvir–velpatasvir will be available locally through a partnership with a generic manufacturer.

From a public health standpoint, having a highly effective treatment that requires minimal diagnostic testing and on-treatment monitoring would be advantageous for reaching large number of patients with limited resources and will be critical achieving the goal of HCV elimination as set forth by the World Health Organization [17].

In summary, results from this Phase 3 study in India suggest treatment with the pangenotypic regimen sofosbuvir–velpatasvir for 12 weeks is safe and effective when administered with minimal monitoring, which has important implications for treating patients in resource-limited regions.

## Abbreviations

AASLD: American Association for the Study of Liver Diseases
APRI: AST:platelet ratio index
BMI: Body mass index
DAA: Direct-acting antiviral
FDA: Food and Drug Administration
HCV: Hepatitis C virus
LLOQ: Lower limit of quantification
RAS: Resistance-associated substitution
SOF: Sofosbuvir
SVR: Sustained virological response
ULN: Upper limit of normal
VEL: Velpatasvir

## References

[1] Puri P, Anand AC, Saraswat VA (2014). Consensus statement of HCV task force of the Indian National Association for study of the liver (INASL). Part I: status report of HCV infection in India. J Clin Exp Hepatol.

[2] Gower E, Estes CC, Hindman S, Razavi-Shearer K, Razavi H (2014). Global epidemiology and genotype distribution of the hepatitis C virus. J Hepatol.

[3] Chowdhury A, Santra A, Chaudhuri S (2003). Hepatitis C virus infection in the general population: a community-based study in West Bengal, India. Hepatology.

[4] Sood A, Sarin SK, Midha V (2012). Prevalence of hepatitis C virus in a selected geographical area of northern India: a population based survey. Indian J Gastroenterol.

[5] Sachdeva S, Mehta B (2012). Population-based hepatitis C survey in a rural block. N Am J Med Sci.

[6] Singh M, Kotwal A, Gupta RM, Adhya S, Chatterjee K, Jayaram J (2010). Sero-epidemiological and behavioural survey of HIV, HBV and HCV amongst Indian armed forces trainees. Med J Armed Forces India.

[7] Chadha MS, Tungatkar SP, Arankalle VA (1999). Insignificant prevalence of antibodies to hepatitis C in a rural area of western Maharashtra. Indian J Gastroenterol.

[8] Olithselvan A, Rela M (2013). Prevalence of hepatitis B and C in puducherry. J Clin Exp Hepatol.

[9] Khaja MN, Madhavi C, Thippavazzula R (2006). High prevalence of hepatitis C virus infection and genotype distribution among general population, blood donors and risk groups. Infect Genet Evol.

[10] Goossens N, Negro F (2014). Is genotype 3 of the hepatitis C virus the new villain?. Hepatology.

[11] Gondeau C, Pageaux GP, Larrey D (2015). Hepatitis C virus infection: are there still specific problems with genotype 3?. World J Gastroenterol.

[12] Fathi H, Clark A, Hill NR, Dusheiko G (2017). Effectiveness of current and future regimens for treating genotype 3 hepatitis C virus infection: a large-scale systematic review. BMC Infect Dis.

[13] Feld JJ, Jacobson IM, Hézode C (2015). Sofosbuvir and velpatasvir for HCV genotype 1, 2, 4, 5, and 6 infection. N Engl J Med.

[14] Foster GR, Afdhal N, Roberts SK (2015). Sofosbuvir and velpatasvir for HCV genotype 2 and 3 infection. N Engl J Med.

[15] Yu M-L, Chuang W-L (2009). Treatment of chronic hepatitis C in Asia: when East meets West. J Gastroenterol Hepatol.

[16] Weins BL, Lystig TC, Berry SM (2013). Recent statistical contributions to medical device development. Therap Innovat Regul Sci.

[17] World Health Organization. Combating hepatitis B and C to reach elimination by 2030. May 2016. Available at: http://apps.who.int/iris/bitstream/10665/206453/1/WHO_HIV_2016.04_eng.pdf?ua=1. Accessed 17 May 2018.

